# Green silver nanoparticles: Prospective nanotools against neurodegenerative cell line model

**DOI:** 10.1002/ibra.12157

**Published:** 2024-05-23

**Authors:** Valeria De Matteis, Simona Martano, Paolo Pellegrino, Chiara Ingrosso, Daniele Costa, Stefano Mazzotta, Jose L. Toca‐Herrera, Rosaria Rinaldi, Mariafrancesca Cascione

**Affiliations:** ^1^ Department of Mathematics and Physics “Ennio De Giorgi” University of Salento Lecce Italy; ^2^ CNR Institute for Microelectronics and Microsystems (IMM) Lecce Italy; ^3^ CNR‐IPCF S.S. Bari, c/o Department of Chemistry University of Bari Aldo Moro Bari Italy; ^4^ Studio Effemme‐Chimica Applicata Squinzano LE Italy; ^5^ Department of Bionanoscience, Institute of Biophysics University of Natural Resources and Life Sciences Vienna (BOKU) Vienna Austria

**Keywords:** nanotool, neurodegenerative diseases, green silver nanoparticles

## Abstract

Neurodegenerative diseases represent an increasingly burdensome challenge of the past decade, primarily driven by the global aging of the population. Ongoing efforts focus on implementing diverse strategies to mitigate the adverse effects of neurodegeneration, with the goal of decelerating the pathology progression. Notably, in recent years, it has emerged that the use of nanoparticles (NPs), particularly those obtained through green chemical processes, could constitute a promising therapeutic approach. Green NPs, exclusively sourced from phytochemicals, are deemed safer compared to NPs synthetized through conventional chemical route. In this study, the effects of green chemistry‐derived silver NPs (AgNPs) were assessed in neuroblastoma cells, SHSY‐5Y, which are considered a pivotal model for investigating neurodegenerative diseases. Specifically, we used two different concentrations (0.5 and 1 µM) of AgNPs and two time points (24 and 48 h) to evaluate the impact on neuroblastoma cells by observing viability reduction and intracellular calcium production, especially using 1 µM at 48 h. Furthermore, investigation using atomic force microscopy (AFM) unveiled an alteration in Young's modulus due to the reorganization of cortical actin following exposure to green AgNPs. This evidence was further corroborated by confocal microscopy acquisitions as well as coherency and density analyses on actin fibers. Our in vitro findings suggest the potential efficacy of green AgNPs against neurodegeneration; therefore, further in vivo studies are imperative to optimize possible therapeutic protocols.

## Graphical abstract

Green nanoparticles (NPs) sourced from phytochemicals are considered safer than conventionally synthesized NPs. This study evaluated the impact of green chemistry‐derived silver NPs (AgNPs) on neuroblastoma cells, a crucial model for neurodegenerative disease research. Results indicate a toxic effect on neuroblastoma cells, affecting calcium levels, vitality, and morphology. Atomic force microscopy revealed an altered Young's modulus, suggesting the potential efficacy of green AgNPs against neurodegeneration, warranting further in vivo studies for therapeutic optimization.

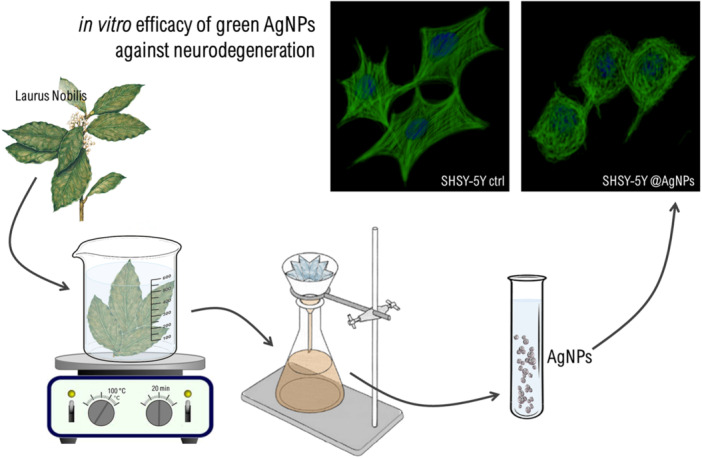

## 1 INTRODUCTION

Neurodegenerative diseases (NDs) represent a heterogeneous group of chronic or possibly hereditary illnesses, primarily caused by degenerative processes that predominantly affect neurons, progressively altering both their structure and functionality. However, these processes may also involve various cells in the immune system. Notable, among these disorders are Parkinson's disease (PD), Alzheimer's disease (AD), Huntington's disease (HD), and amyotrophic lateral sclerosis (ALS). While these pathologies exhibit distinct pathophysiological properties, they are concurrently linked as neuroinflammation, neuronal loss, misfolded protein aggregation, oxidative stress, and autophagy dysregulation.^1^ These pathologies demonstrate selective vulnerability of neurons, coupled with the degeneration of specific brain regions. Additionally, an abnormal deposition of proteins, either extracellular or intracellular, occurs in neurons or other types of brain cells.^2^

Current therapeutic approaches, commonly administered orally or intravenously, are only capable of alleviating the severity of symptoms for their constrained ability to overcome the blood–brain barrier (BBB) and reach the central nervous system.^3^ However, the utilization of NPs has emerged as a potent approach to enhance the penetration of bioactive molecules through the BBB^4^, ^5^ due to their unique physicochemical properties.^6^

Furthermore, as documented in the literature, various nanosystems exhibit efficacy against neuroinflammation, protein aggregation, and oxidative stress in NDs, encompassing both organic and inorganic NPs.^7^, ^8^, ^9^, ^10^ The therapeutic potential for damage recovery and alleviation of neuronal inflammation has been evaluated in the treatment of AD and PD, particularly with noble metal NPs (gold NPs [AuNPs] and silver NPs [AgNPs]).^11^ These NPs have demonstrated a substantial improvement in damaged neurons, attributed to their anti‐inflammatory and antioxidant properties.^12^ The intriguing physicochemical features of AgNPs, encompassing tunable optical properties, straightforward synthesis, and surface functionalization, allow their versatile application as nanotools in medicine.^13^ In comparison to traditional therapeutics, these appealing nanomaterials can be easily tracked within the human body, displaying enhanced penetration through the BBB and thus promoting a significant brain immune response. The growing demand for AgNPs in nanomedicine and the broader biomedical field has led to the shift from chemical to more environmentally friendly approaches for nanomaterial synthesis.^14^ In the classical chemical routes, AgNPs were synthesized using hazardous chemicals and harmful reducing and capping agents, raising serious concerns over their toxicity.^15^ Alternatively, a green approach can be applied to obtain monodispersed and highly stable NPs with reduced toxicity.^16^, ^17^, ^18^ The reduction of metal ions and the prevention of undesired agglomeration phenomena can be facilitated through the role of green catalysts (e.g., polyphenols, tannins, vitamins, saponins, enzymes) naturally present in the green raw starting materials. This ensures simple, reliable, and efficient eco‐friendly processes with low environmental impact.^19^, ^20^

In our work, we initially synthesized AgNPs with a size of 30 nm using *Laurus nobilis* leaf extract. This plant is widespread in the Mediterranean area, and its leaves have demonstrated high antioxidant activity due to high levels of polyphenols and terpenes, which serve as both reducing and capping molecules. The NPs obtained from this plant are reproducible and allow for high yield starting from a small amount of biomass. Subsequently, the cytotoxic effect was assessed on neuroblastoma cell line SHSY‐5Y, a suitable model for exploring the pathophysiological mechanisms of NDs. SHSY‐5Y were exposed at two different concentrations of AgNPs (0.5 and 1 µM) over two different time points (24 and 48 h), and the viability and calcium levels have been quantified. Confocal laser scanning microscopy (CLSM) and atomic force microscopy (AFM) were employed to evaluate the effects on actin fibers organization followed by Young's modulus measurements. The obtained results suggest the capability of green AgNPs to induce adverse effects in the SHSY‐5Y cell line, thus positioning them as a promising nanotool in the therapeutic approach against NDs.

## 2 MATERIALS AND METHODS

### 2.1 Synthesis of AgNPs

AgNPs have been synthetized following the green route protocol as previously described in Cascione et al.^20^ Leaves of *L. nobilis* were collected, washed with MilliQ to remove pollutants and contaminants, and then air‐dried at room temperature for 1 day. Subsequently, 10 g of leaves were placed into a glass flask with 100 mL of MilliQ water (1:10 ratio) and boiled at 100°C for 20 min. The mixture was cooled, and the extract was filtered before it was used for NP synthesis. A measure of 2.5 mL of leaf extracts was added to 50 mL of AgNO_3_ (1 mM) and heated to 60°C for about 45 min. Then, the reaction color switched from light yellow to deep brown, indicating the chemical reduction of Ag^+^ ions into Ag^0^ (pH 7). Finally, solutions were moved to centrifuge tubes and centrifuged at 4000 rpm for 1 h to achieve NPs. NPs were further purified with MilliQ water using three cycles of centrifugation conducted at 13,000 rpm. After they were resuspended in water, the concentrations of 0.5 and 1 µM of AgNPs were obtained.

### 2.2 NP characterization

Transmission electron microscopy (TEM) analyses were conducted using a Jeol Jem‐1011 microscope operating at 100 kV. The instrument was equipped with a high‐contrast objective lens that provides an ultimate point resolution of 0.34 nm, and the images were captured with the Quemesa 138 Olympus CCD 11 Mp camera. The samples for TEM observations were prepared by depositing ~10 µL of the aqueous solution containing the metallic NPs onto carbon‐coated grids.

Dynamic light scattering (DLS) and *ζ*‐potential measurements for AgNPs in aqueous solutions (25°C, pH 7) were recorded by a Zetasizer Nano‐ZS equipped with a HeNe laser (633 nm, 4.0 mW) and a detector (ZEN3600; Malvern Instruments Ltd.). DLS measurements are based on the fluctuations in light intensity analysis over a period of time, caused by the Brownian motion of particles, to determine the diffusion coefficient (*D*). This coefficient is linked to the hydrodynamic radius (*R*_h_) of the particle by the Stokes–Einstein equation:

$$D=\frac{k_{b}T}{6\pi \eta R_{h}},$$

where *κ*_b_ is the Boltzmann constant (1.380 × 10^−23^ kg m^2^ s^−2^ K^−1^), *T* is the absolute temperature, and *η* is the viscosity of the medium.^21^ *ζ*‐Potential is determined by measuring the electrophoretic mobility of charged particles when subjected to an applied electric field. The electrophoretic mobility (*µ*_e_) of the particles is calculated using Henry's equation:

$${µ}_{e}\frac{2\varepsilon_{r}\varepsilon_{0}\zeta f(K_{a})}{3\eta },$$

where *ε*_r_ is the relative permittivity/dielectric constant, ε_0_ is the permittivity of vacuum, *ζ* is the *ζ*‐potential value, *f* (*K*_a_) is the Henry's or Helmholtz–Smoluchowski function, and *η* is the viscosity at the experimental temperature.^22^ The ultraviolet–visible (UV–vis) spectroscopy analysis in the spectral range of 300–800 nm was performed at room temperature using a Varian Cary 5 spectrophotometer (ZEN3600; Malvern Instruments Ltd.) equipped with a quartz cuvette having 10 mm path.

### 2.3 Determination of Ag concentration

The green Ag concentrations were calculated by elemental analyses using inductively coupled plasma‐optical emission spectroscopy (ICP‐OES) PerkinElmer AVIO 500. A total of 150 µL of the NP solutions were digested overnight, by adding 1.5 mL of HNO_3_, followed by dilution with MilliQ water (1:10).

### 2.4 Cell culture

Human neuroblastoma SHSY‐5Y cells (ATCC® CRL‐2266™) were maintained in Dulbecco's modified Eagle's medium (DMEM) (Sigma‐Aldrich) enriched with 1% of glutamine, 1% of penicillin/streptomycin (Sigma‐Aldrich), and 10% of fetal bovine serum (Sigma‐Aldrich). Cells have been grown in a humidified controlled atmosphere with a 95% to 5% ratio of air/CO_2_ at 37°C.

### 2.5 Cell viability assessment

SHSY‐5Y were seeded at a concentration of 5 × 10^3^ cells/well in 96‐well plates and stabilized in a humidified controlled atmosphere with a 95%–5% ratio of air/CO_2_ at 37°C for 24 h. Subsequently, green AgNPs at two different concentrations (0.5 and 1 µM) were added to cell media. After incubation times of 24 and 48 h, standard WST‐8 assay (96992; Sigma‐Aldrich), following the procedure previously described,^23^ was used. WST‐8 is one of the water‐soluble tetrazolium salts (WST) [2‐(2‐methoxy‐4‐nitrophenyl)‐3‐(4‐nitrophenyl)‐5‐(2,4‐disulfophenyl)‐2*H*‐tetrazolium]. The control tests were performed on SHSY‐5Y incubated with equivalent volumes grown solution of 10% (v/v) dimethyl sulfoxide (Sigma‐Aldrich) in Dulbecco's phosphate‐buffered saline (DPBS). Samples were measured by using a Fluo Star Optima (BMG LABTECH) microplate reader at a wavelength of 460 nm. Data were collected by Control Software and elaborated with MARS Data Analysis Software (BMG LABTECH). The data, obtained on eight different viability experiments, were expressed as mean ± SD.

### 2.6 Intracellular calcium level determination

SHSY‐5Y cells were seeded at a concentration of 7 × 10^4^ cells/mL in glass Petri dishes. After 24 h of stabilization, the culture media were removed and replaced with fresh culture media enriched with 0.5 and 1 µM of green Ag NPs. After incubation at 24 and 48 h, the samples were homogenized by sonication in 100 µL of lysis buffer (100 mM Tris, pH 7.5) to quantify the intracellular Ca^2+^ amount. The homogenates were centrifuged at 10^5^ *g* for 15 min at 4°C. The supernatant was collected and stored on ice; the samples were analyzed on the same day using a Ca^2+^ Assay Kit (ab102505; Abcam), following the manufacturer's instructions.

### 2.7 Confocal analysis of actin fibers

SHSY‐5Y cells were seeded in a 24‐well plate at a concentration of 10^5^ cells/well and then incubated with green AgNPs for 24 and 48 h at two concentrations (0.5 and 1 µM). After NP exposure, the medium was removed, and cells were gently washed with PBS and successively fixed with 0.25% glutaraldehyde (Merck KGaA) (v/v) for 20 min. Finally, cells were permeabilized with 0.1% Triton X (Merck KGaA) (v/v) for 10 min. The actin fibers were stained with FITC‐phalloidin (Merck KGaA) at a concentration of 1 µg/mL overnight. Laser scanning confocal microscopy acquisition was obtained using a Zeiss LSM700 (Zeiss) confocal microscope equipped with an Axio Observer Z1 (Zeiss) inverted microscope using ×100, 1.46 numerical aperture oil immersion lens for imaging. Confocal data were processed using ZEN2010 software (Zeiss). Morphometric quantification (coherency and density of F‐actin) was performed on 25 cells using ImageJ 1.47 open‐source software calculator.

### 2.8 Atomic force spectroscopy characterization

Indentation force curves were recorded by using a Bioscope Catalyst AFM (Bruker Inc.) operating in force–volume (FV) mode. All experiments were performed using Silicon Nitride V‐shaped Bruker's Sharp Microlever (MSNL, tip C; Bruker Inc.), with a nominal spring constant of 0.01 N/m, which was determined with high precision, according to the thermal tune method, before performing each measurement. The FV mapping was acquired 50 µm∗50 µm areas, setting the acquisition parameters for all experiments as follows: ramp rate 4.88 Hz, FV scan rate 0.03 Hz, and trigger threshold 50 nm. The resolution of the FV topography acquisition channel was fixed at 64 (sample per line) × 64 (lines) to reduce the acquisition time and avoid deterioration of the living sample but it is still able to visualize the cell bodies. The latter condition was crucial to distinguish the mechanical response of the cellular membrane in correspondence to the nuclear or cytoplasmic region. In this aim, 20 force–distance (FD) curves were manually selected on the cytoplasmic area; then, they were analyzed by Nanoscope Analysis software (Bruker Inc.) to extract Young's modulus values using the procedure described in previous works.^24^, ^25^, ^26^ The analysis was performed on 20 different cells for each treatment; therefore, Young's modulus was expressed as mean value ± SD.

### 2.9 Statistics analysis

All experimental data were analyzed and plotted by using Origin Pro v8 (Origin‐Lab Corporation). The differences between the two groups were calculated by a two‐tailed Student's *t*‐test. The comparison between three and more groups was analyzed by one‐ or two‐way analysis of variance multiple comparisons, respectively. The differences were statistically significant when *p < 0.05.

## 3 RESULTS AND DISCUSSION

Over the past few decades, the commercial demand for NPs has witnessed rapid growth across various fields, including industrial processes, electronics, environmental technology, energy, and, notably, biomedicine. However, the increasing production and application of NPs inherently pose potential hazards to the environment and living organisms. Consequently, contemporary efforts within the scientific community are focused on developing eco‐friendly synthetic protocols. These protocols aim to optimize the production of NPs with reduced toxicity by substituting conventional chemical reagents with phytochemical compounds.

**Figure 1:**
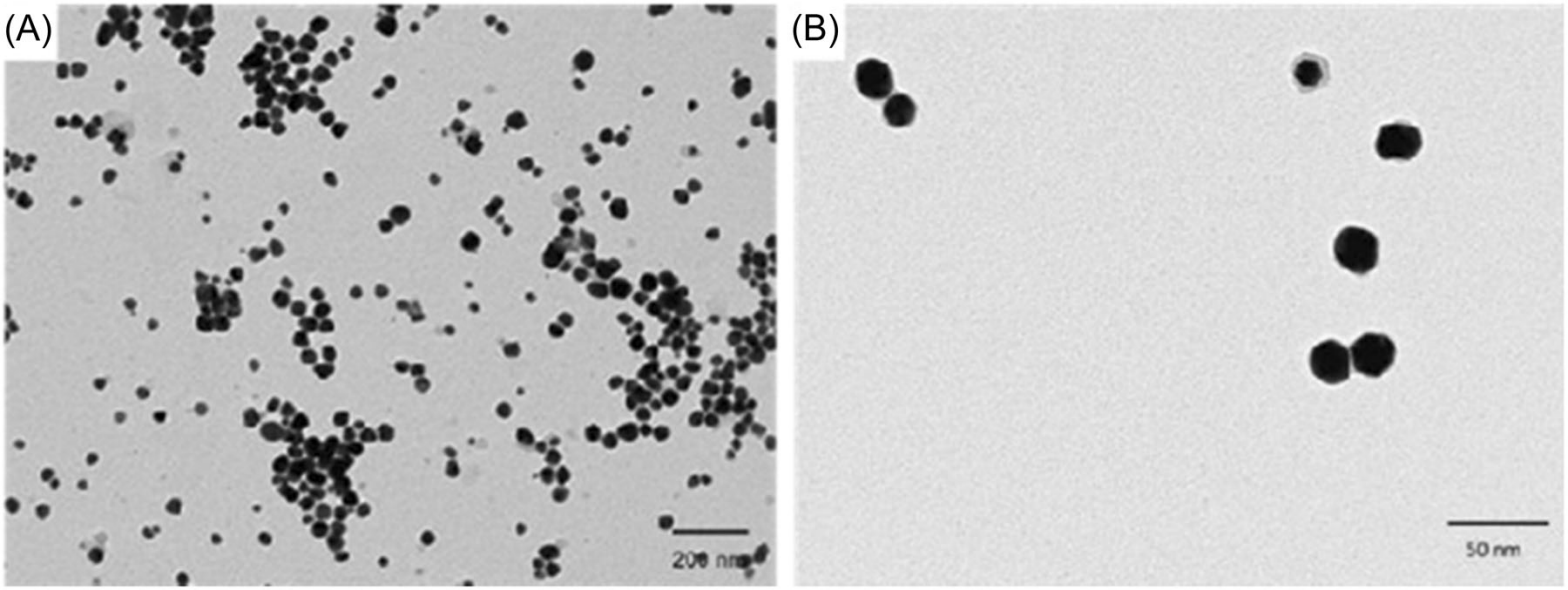
Representative transmission electron microscopy image of green silver nanoparticles. Scale bar 200 nm (A) and 50 nm (B).

In this context, we utilized plant waste, specifically *L. nobilis*, to obtain stable AgNPs in the aqueous solution. The synthesis process was reproducible, enabling the production of high concentrations of NPs, confirming as reported in our previous work.^20^ The AgNP morphology observed through TEM acquisition (Figure 1A,B) appeared to be almost spherical, with a diameter of approximately 28 nm. For a more comprehensive characterization of these nanostructures, their dimensions were further confirmed through DLS analysis, yielding a value of 30 ± 5 nm. Additionally, given that the polyphenols and other molecules present in the plant extract serve not only as reducing agents but also as capping agents, the surface charge was negative, as shown by the *ζ*‐potential value (−30 ± 6 mV) (Table 1). The *ζ*‐potential refers to the electrostatic potential that exists at the shear plane, or the boundary between a charged surface and the liquid medium in which it is dispersed. In general, *ζ*‐potential plays a crucial role in determining the stability of colloidal dispersions. Particles with high *ζ*‐potentials repel each other strongly and are less likely to aggregate or flocculate, resulting in a stable dispersion. Conversely, particles with low *ζ*‐ potentials may tend to aggregate, leading to instability and eventual precipitation. Then, our NPs appeared to be stable in water. To assess the stability of AgNPs in a cell culture medium, DLS measurements were conducted in DMEM. As expected, the NPs increased in size due to the formation of protein corona on their surface, resulting in a size of 35 ± 7 nm. Then, we moved to assess the *ζ*‐potential of AgNPs showing changes due to the significant presence of serum proteins on the surface, becoming more negative (−41 ± 6 mV).

**Table 1:** DLS and *ζ*‐potential values of green AgNPs were measured in an aqueous solution (pH 7) and in a cell culture medium.

	DLS	DLS in DMEM	*ζ*‐Potential	*ζ*‐Potential in DMEM
Green AgNPs	30 ± 5 nm	35 ± 7 nm	−30 ± 6 mV	−41 ± 6 mV

Abbreviations: AgNPs, silver nanoparticles; DLS, dynamic light scattering; DMEM, Dulbecco's modified Eagle's medium.

**Figure 2:**
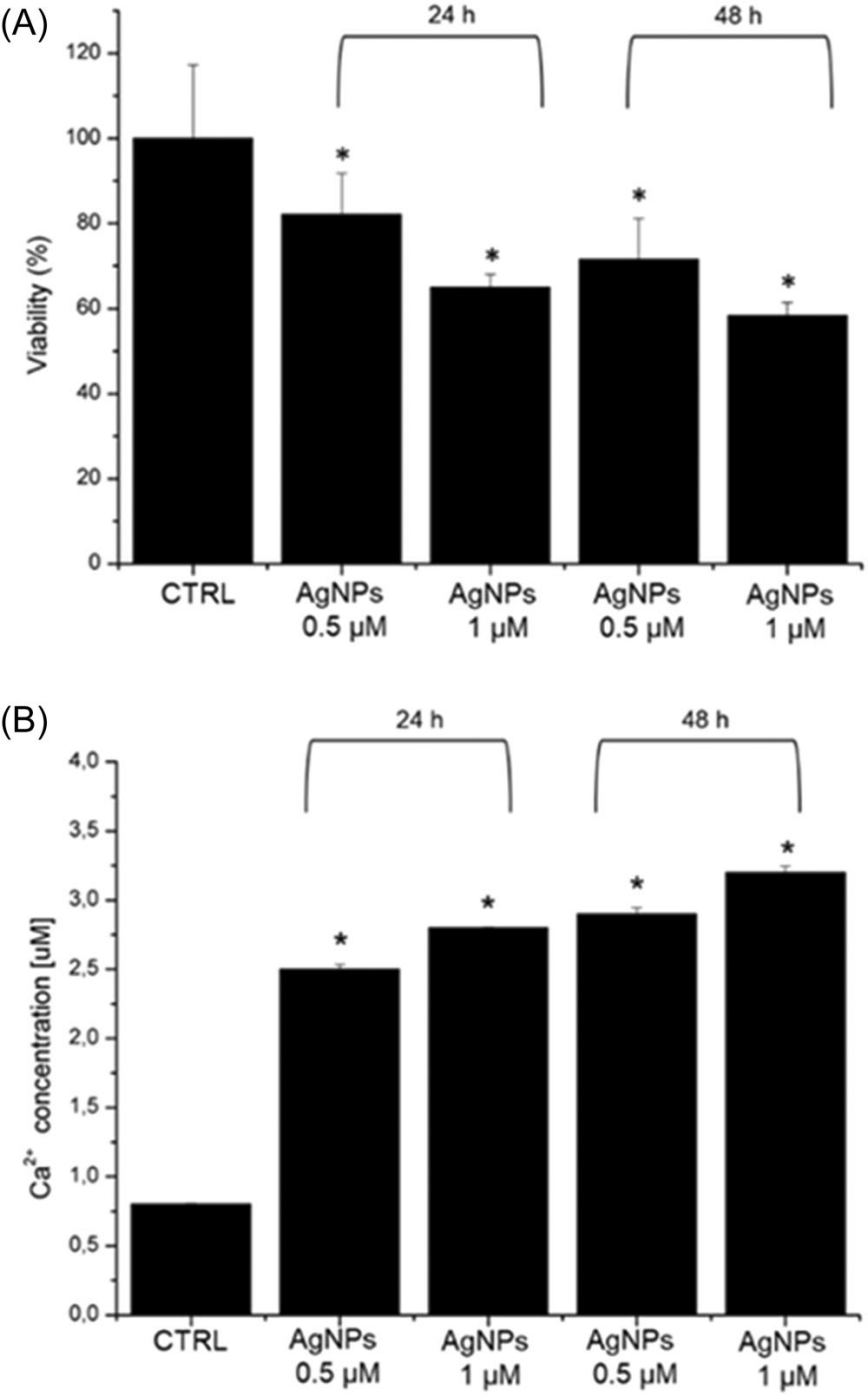
(A) Viability assay (WST‐8) of SHSY‐5Y cell lines after 24 h and 48 h of exposure to two doses of green AgNPs. The viability of NP‐treated cells was normalized to non‐treated control cells. As a positive control (P), the cells were incubated with 5% DMSO (data not shown). Data reported as the mean ± SD from three independent experiments are considered statistically significant, compared with the control (*n* = 8) for *p* value < 0.05 (<0.05 *). (B) Effect of green AgNPs on the Ca^2+^ level in SHSY‐5Y cells after 24 h and 48 h of exposure to two doses of green AgNPs following the procedure described in the section materials. The data are reported as the mean ± SD from three independent experiments; *p < 0.05, compared with control (*n* = 8).

Following the morphological characterization of the NPs, we evaluated their impact on reducing viability in SHSY‐5Y cells using two concentrations: 0.5 and 1 µM over 24 and 48 h. The results obtained through the WST‐8 assay (Figure 2A) revealed that the AgNPs induced a dose‐ and time‐dependent decrease in cell viability. Specifically, after 24 h of exposure, the vitality rate decreased by 18% and 34% with the lower and higher concentrations of AgNPs, respectively. These percentages were significantly reduced extending the treatment duration to 48 h, reaching 35% and 42% mortality. Subsequently, we assessed intracellular Ca^2+^ levels, a ubiquitous intracellular signaling cation that regulates numerous cellular processes. Typically, a small quantity of Ca^2+^ is present within the mitochondrial/nuclear matrix and cytosol, while elevated concentrations are in the endoplasmic reticulum, muscle cells, and the sarcoplasmic reticulum.^27^ Disrupting of this equilibrium by stimuli leads to an increase in cytosolic Ca^2+^ concentration. Under such circumstances, apoptosis and necrosis may occur.^28^ In our study, we observed an increase in calcium levels when AgNPs were used to expose cells (Figure 2B), suggesting the homeostasis alteration of Ca^2+^. In particular, using the concentration of 0.5 µM of AgNPs, 2.5 µM of Ca^2+^ was observed compared to the control cells (0.75 µM) after 24 h of exposure; the Ca^2+^ amount slightly increased after 48 h, becoming equal to 2.7 µM. A similar but more pronounced trend was observed when exposing SHSY‐5Y cells to AgNPs at a concentration of 1 µM. Specifically, the calcium concentration measured 2.8 and 3.3 µM after exposure periods of 24 and 48 h, respectively.

To understand if the difference in viability and calcium level amount could be related to different uptake levels, we quantify the NP engulfment by ICP‐EOS over lysed cells. As shown in Table 2, the Ag concentration increased in a time‐ and dose‐dependent manner.

**Table 2:** Ag accumulation in SHSY‐5Y cell lines.

Group	Ctrl	0.5 µM of AgNPs	1 µM of AgNPs
24 h	0 µg	3.2 ± 0.5 µg	4.5 ± 0.9 µg
48 h	0 µg	4.3 ± 0.8 µg	4.8 ± 0.7 µg

*Note*: The Ag accumulation in SHSY‐5Y cell lines was exposed to 0.5 and 1 µM of AgNPs after 24 and 48 h of exposure. The control was represented by untreated cells (values = 0).

Abbreviations: AgNP, silver nanoparticle; Ctrl, control.

**Figure 3:**
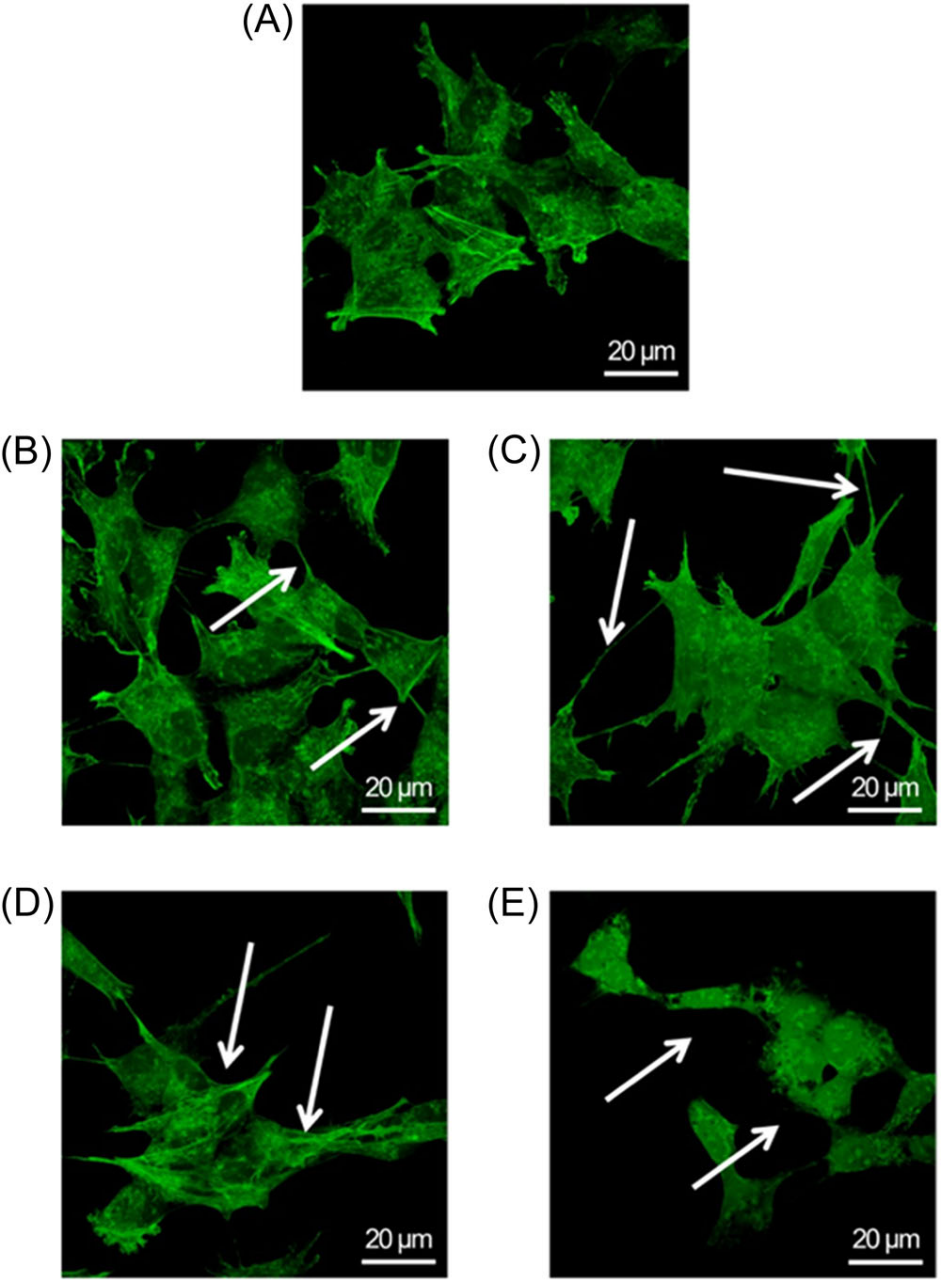
Confocal images of SHSY‐5Y. Representative confocal images of nontreated cells (A), cells exposed to 0.5 μM green silver nanoparticles(AgNPs) for 24 h (B) and 48 h (C), and cells exposed to 1 μM green AgNPs for 24 h (D) and 48 h (E). The cells were fixed and then stained with phalloidin‐FITC. Two‐dimensional images of cortical actin were acquired using a Zeiss LSM700 (Zeiss) confocal microscope, equipped with an Axio Observer Z1 (Zeiss) inverted microscope, using a ×100, 1.46 numerical aperture oil immersion lens. All data were processed using the ZEN software (Zeiss). The white arrows showed structural alterations. Scale bar: 20 µm. [Color figure can be viewed at wileyonlinelibrary.com]

Following the cytotoxicity assessment, we proceeded to investigate the potential alteration of the cytoskeleton, specifically focusing on the organization of the cortical actin, through CLSM acquisitions (Figure 3). Assessing the remodeling of cortical actin meshwork in treated cells with respect to untreated ones is crucial, as it can impact cellular mechanics behavior. Accordingly, we labeled actin fibers with FITC after incubating the cells for 24 and 48 h at the two concentrations of AgNPs obtained through green synthesis. In the control group (Figure 3A), the SHSY‐5Y cells displayed the characteristic appearance of neuroblast‐like cells, featuring neurites extending in their vicinity. Furthermore, the actin filaments exhibited a high degree of organization, and the cells were interconnected. Exposure to green AgNPs at the two concentrations employed in the preceding tests resulted in different outcomes. Specifically, at the lower concentration and after 24 h (Figure 3B), the cell essentially maintained their morphology but exhibited more pronounced neurites, which maintain the connection with the nearest cells. These effects became more pronounced with prolonged exposure time until 48 h (Figure 3C): the cell body does not undergo significant changes in its shape but appears to increase its surface area. The cells become more distant from each other, yet they maintain contact through a pronounced elongation of neurite‐like filaments. At a concentration of 1 µM, the cells experienced a substantial change in their shape after an exposure time of 24 h (Figure 3D), manifesting a more elongated cellular structure and a disruption of the cortical actin organization, which seemed to be preserved only locally. After 48 h of exposure, the F‐actin fibers appeared completely defragmented, with cells exhibiting detachment and loss of morphology (Figure 3E). The aforementioned speculations, solely derived from a qualitative assessment of high‐resolution fluorescence images, were corroborated by quantitative measurements obtained through the analysis of density and coherency parameters: the fluorescence density observed in actin‐stained images functions as a direct indicator of the local actin concentration, while coherency offers insights into the network organization of fibers by estimating the local orientation within small image regions.^29^

**Figure 4:**
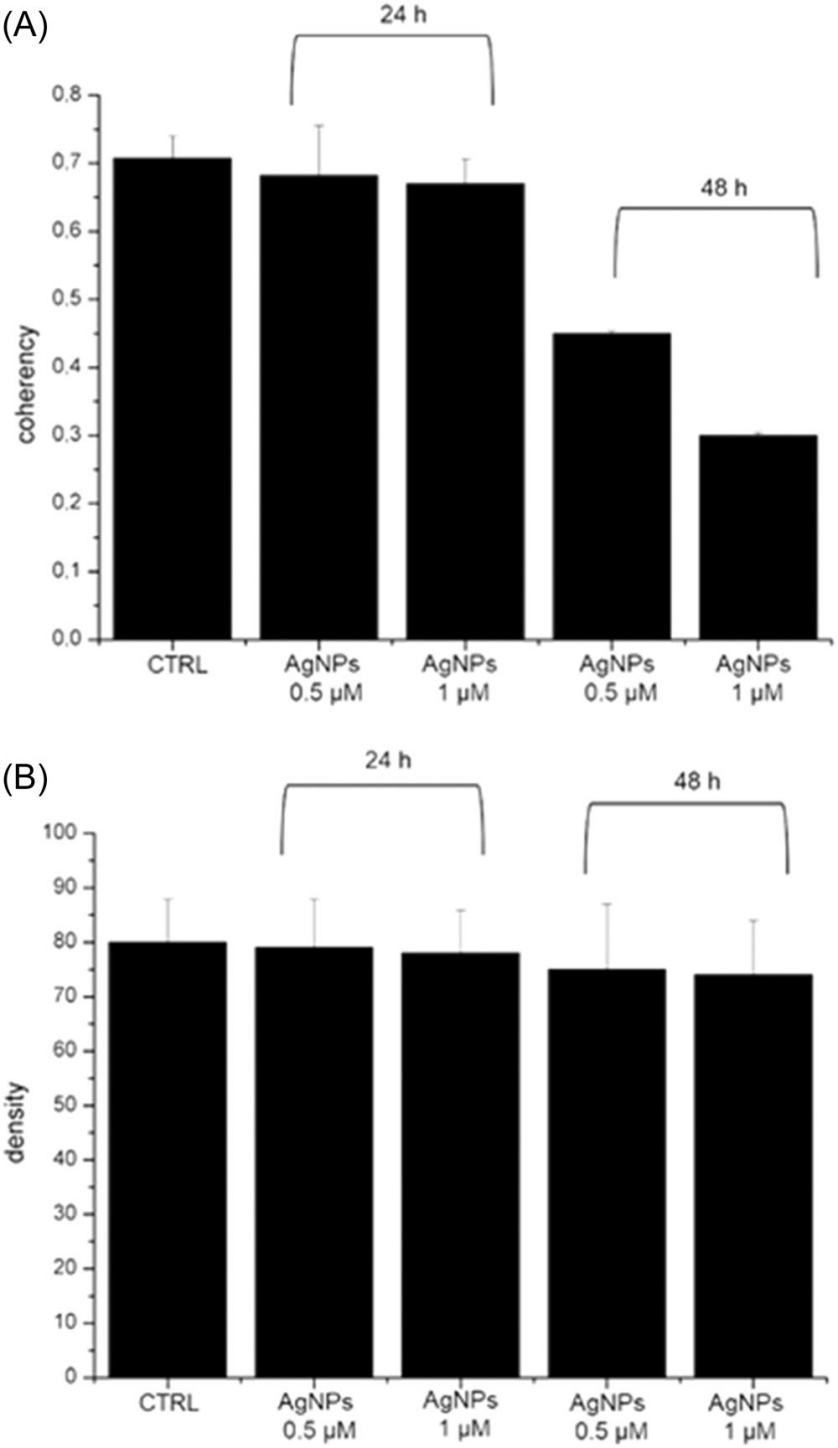
Coherency (A) and density (B) values of SHSY‐5Y cells acquired by ImageJ software on 15 cells. AgNP, silver nanoparticles; CTRL, control.

The obtained results (Figure 4) illustrate a time‐ and concentration‐dependent decrease in the coherency (Figure 4A) and density (Figure 4B) values. The coherency value obtained for the control sample, equal to 0.71 ± 0.04, does not undergo a significant variation after 24 h of exposure to green AgNPs at both 0.5 and 1 μM concentrations. However, although it did not exhibit statistical difference, extending the exposure time to 48 h resulted in a change in the coherency value, amounting to 26% and 39% for concentrations of 0.5 and 1 μM, respectively. Differently, the density level appeared consistent for both control cells and all treated cases. Therefore, the analysis of fluorescence acquisitions performed on the actin network indicated that the actin amount remained constant, but its organization of actin was severely compromised, consequently affecting the signaling of mechanical stimuli. This experimental evidence showed that the green AgNPs were safe using low concentration and short time on neuroblastoma cells. However, when the concentration increased, some toxic effects were reported, confirming the trend recorded in the biological tests (Figure 2).

**Figure 5:**
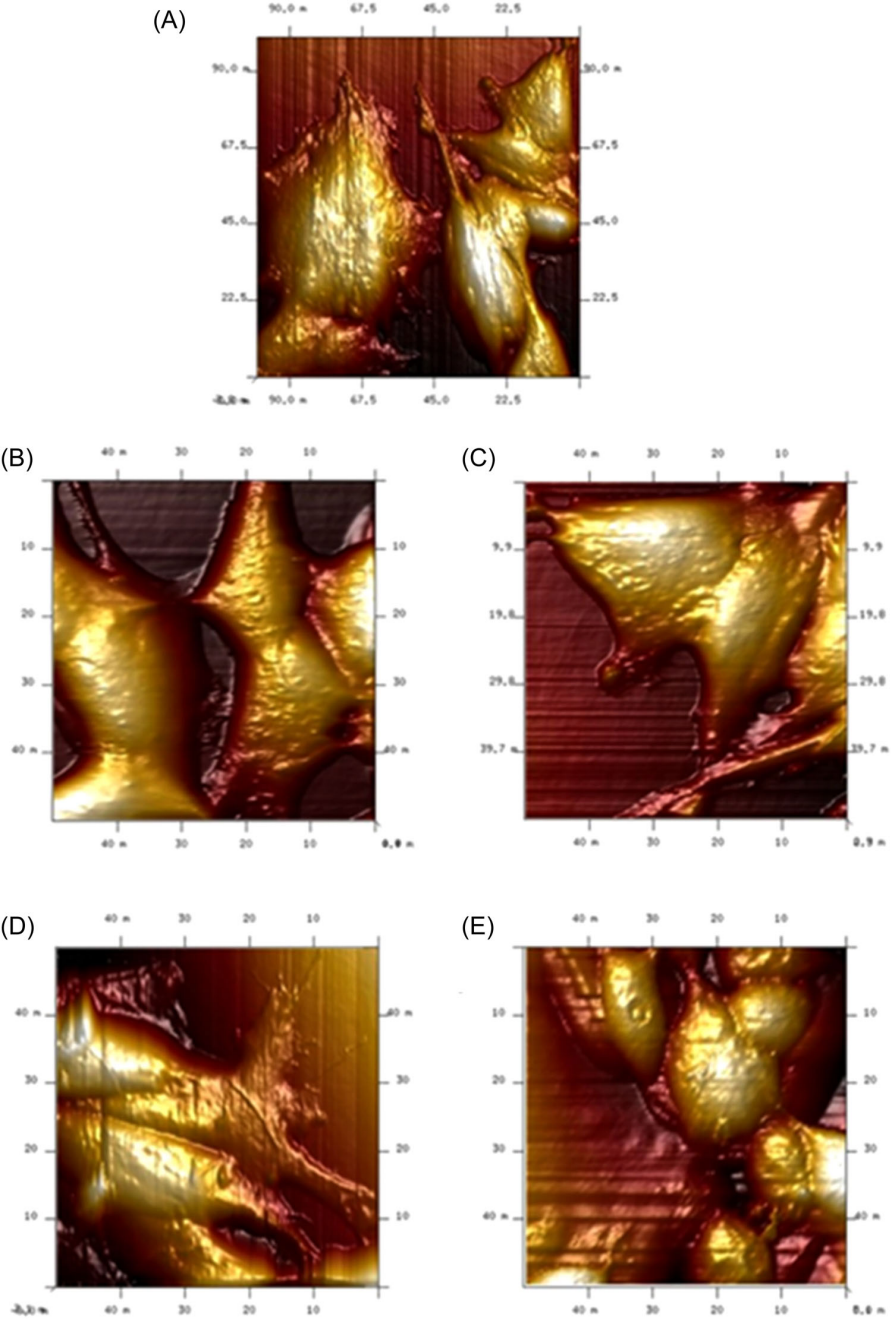
Representative atomic force microscopy images of SHSY‐5Y. Control cells (A), cells exposed to 0.5 μM green silver nanoparticles (AgNPs) for 24 h (B) and 48 h (C), and cells exposed to 1 μM green AgNPs for 24 h (D) and 48 h (E). [Color figure can be viewed at wileyonlinelibrary.com]

Cellular morphology was further investigated using AFM. Topographical acquisitions performed on areas of 50 μm × 50 μm enabled visualization of submicrometric structures present on the cell surface **(**Figure 5). As observed in confocal acquisitions, we noted the same cell morphology in the control and treated samples. Specifically, the untreated cells appeared to be regular, and distinct protrusions were found indicating the presence of an organized actin structure (Figure 5A). The treatment at 24 h with two concentrations (Figure 5B,D) and 48 h (Figure 5C,E) induced alterations that were time‐ and dose‐dependent.

Considering that the rearrangement of the cortical actin inevitably leads to a variation in the elastic behavior of the cell, we quantified this in terms of Young's modulus parameter.^30^ Specifically, variations in this parameter can be regarded as an indicative marker for potential pathological conditions or employed as a predictive parameter for forecasting cellular behavior.^31^

**Figure 6:**
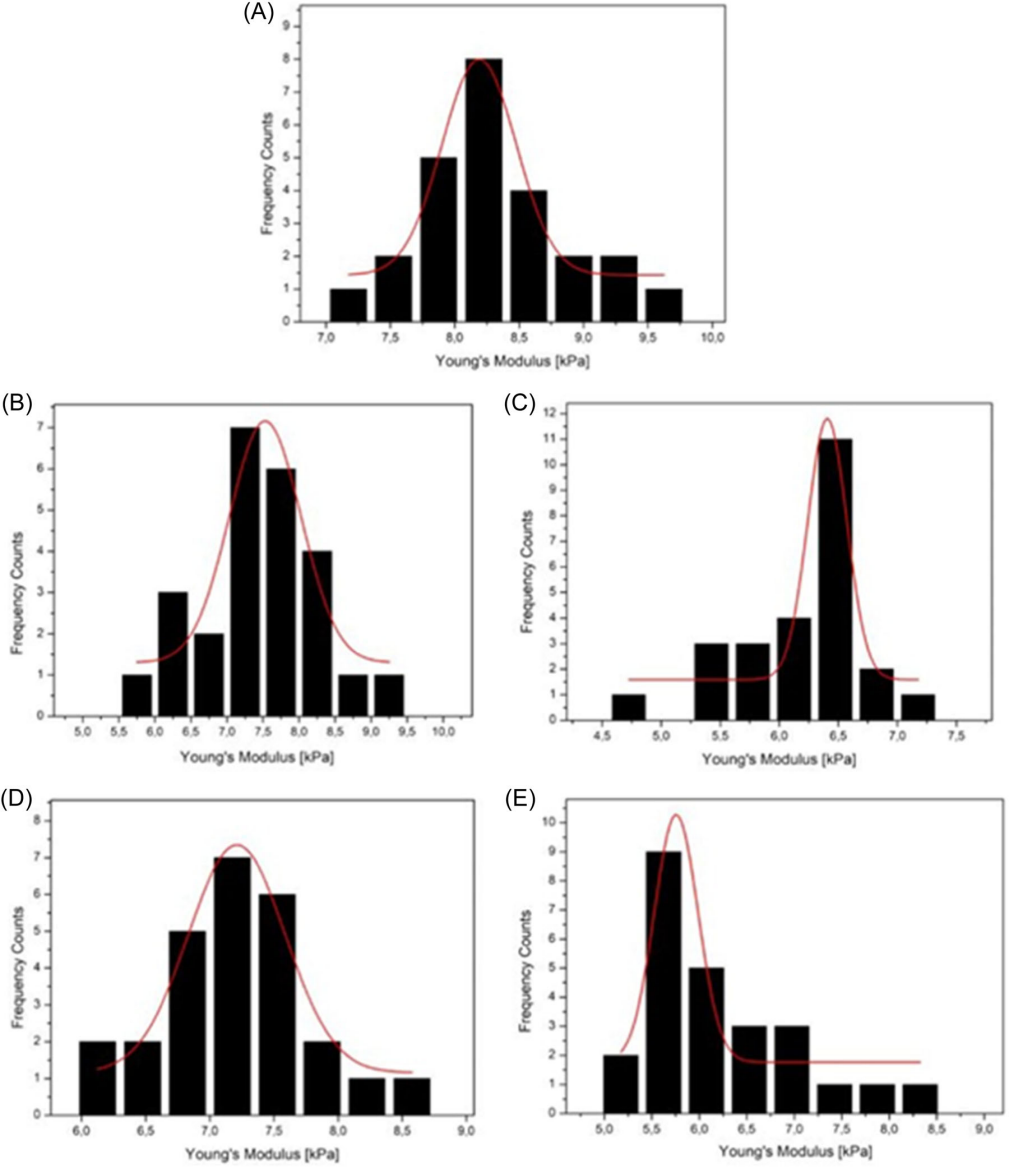
Young's modulus distributions with Gaussian fit functions (red line). Young's modulus distributions in control cells (A), and in cells exposed to green AgNPs at a concentration of 0.5 and 1 µM for 24 h (B, C) and 48 h (D, E), respectively. [Color figure can be viewed at wileyonlinelibrary.com]

In light of the preceding statements, Young's modulus was determinate in correspondence of cytoplasmic cellular area through FD curves acquired by AFM operating FV mode (Figure 6). Control cells (Figure 6A) showed Young's modulus of 8.3 ± 0.3 kPa. Following 24 h exposure to green AgNPs at a concentration of 0.5 µM, a slight decrease of 9% was recorded compared to the control, resulting in a Young's modulus value of 7.5 ± 0.1 kPa (Figure 6B). A more substantial reduction in elasticity parameter, amounting to 12%, was observed when the exposure to NPs was extended to 48 h, leading to a Young's modulus of 7.2 ± 0.1 kPa (Figure 6D). The same trend was noted in SH‐SY5 cells exposed to green AgNPs at a concentration of 1 µM. In this case, the decrease in the elasticity parameter was more noticeable, precisely 22% and 29% after 24 and 48 h of exposure, respectively. Specifically, the obtained Young's modulus for cell membrane elasticity in the cytoplasmic region was 6.4 ± 0.1 kPa after 24 h (Figure 6C) and further declined to 5.7 ± 0.5 kPa after 48 h (Figure 6E). The change in cell membrane elasticity values perfectly aligned with the findings derived from the observation and analysis of confocal microscopy acquisitions. The decrease in Young's moduli could indeed be ascribed to the defragmentation of actin filaments, thereby diminishing the cell's ability to withstand elastic deformation. This alteration also manifested in changes to the cell membrane, encompassing the disruption of sites where the cytoskeleton connects to the cell membrane. Moreover, the reduction in Young's modulus confirmed the dependency on both the time of exposure and the concentration of NPs. These findings suggested a disruptive role of green NPs in neuroblastoma cells, making them a potential tool for applications in neurodegenerative cell models.

## 4 CONCLUSIONS

In conclusion, our study revealed that AgNPs synthetized through the green chemistry paradigm, using *L. nobilis* leaf extract, exhibited toxic properties on neuroblastoma cells, specifically SHSY‐5Y, commonly employed as a model for NDs in the literature. Notably, at low concentrations of NPs, cell viability remains minimally affected; however, significant morphological changes at the cortical level, in terms of both morphology and changes in membrane elasticity, were observed. This suggested that the actin rearrangement may be linked to a modification of the plasma membrane, which becomes softer with higher NP concentrations and longer exposure times. Considering these findings, the use of green AgNPs as a potential counteractive agent against degeneration could be further assessed in subsequent in vivo studies.

## AUTHOR CONTRIBUTIONS

Valeria De Matteis and Mariafrancesca Cascione wrote and drafted the manuscript. Valeria De Matteis, Mariafrancesca Cascione, Simona Martano, Paolo Pellegrino, Chiara Ingrosso, Daniele Costa, and Stefano Mazzotta performed the experiments. Valeria De Matteis, Mariafrancesca Cascione, Paolo Pellegrino, and Daniele Costa analyzed the data. Stefano Mazzotta performed the ICP‐OES analysis. Valeria De Matteis, Mariafrancesca Cascione, Jose Luis Toca‐Herrera, and Rosaria Rinaldi edited the manuscript and supervised the work.

## CONFLICT OF INTEREST STATEMENT

The authors declare no conflict of interest.

## ETHICS STATEMENT

This study does not involve experimental animals and human subjects; therefore, it does not involve ethics.

## Data Availability

The data sets generated and analyzed during the current study are available from the corresponding author upon reasonable request.
